# Grain morphology reconstruction of crystalline materials from Laue three-dimensional neutron diffraction tomography

**DOI:** 10.1038/s41598-020-60330-w

**Published:** 2020-02-28

**Authors:** Stavros Samothrakitis, Marc Raventós, Jan Čapek, Camilla Buhl Larsen, Christian Grünzweig, Michael Tovar, Marina Garcia-Gonzalez, Jaromír Kopeček, Søren Schmidt, Markus Strobl

**Affiliations:** 1 Nuclear Physics Institute of the Czech Academy of Sciences, Hucinec - Řež, čp. 130, 250 68 Řež, Czech Republic; 2 FZU Institute of Physics of the Czech Academy of Sciences, Na Slovance 1999/2, 182 21 Prague, Czech Republic; 3 Neutron Imaging & Applied Materials Group, Laboratory for Neutron Scattering & Imaging, Paul Scherrer Institut, CH-5232 Villigen, PSI Switzerland; 4 University of Geneva, Department of Quantum Matter Physics, 24 Quai Ernest-Ansermet, CH-1211 Geneva 4, Geneva, Switzerland; 5 Helmholtz-Zentrum Berlin, Department Structure and Dynamics of Energy Materials, Hahn-Meitner-Platz 1, 14109 Berlin, Germany; 6 Photons for Engineering and Manufacturing Group, Laboratory for Synchrotron Radiation-Condensed Matter, Paul Scherrer Institut, CH-5232 Villigen, PSI Switzerland; 7 Neutrons and X-rays for Mechanics of Materials Group, IMX, STI École Polytechnique Fédérale de Lausanne, CH-1015 Lausanne, Switzerland; 8 Technical University of Denmark, Department of Physics, Fysikvej, 2800 Kgs. Lyngby, Denmark; 9 Niels Bohr Institute, University of Copenhagen, 2100 Copenhagen, Denmark

**Keywords:** Characterization and analytical techniques, Imaging techniques

## Abstract

The macroscopic properties of advanced engineering and functional materials are highly dependent on their overall grain orientation distribution, size, and morphology. Here we present Laue 3D neutron diffraction tomography providing reconstructions of the grains constituting a coarse-grained polycrystalline material. Reconstructions of the grain morphology of a highly pure Fe cylinder and a Cu cube sample are presented. A total number of 23 and 9 grains from the Fe and Cu samples, respectively, were indexed and reconstructed. Validation of the grain morphological reconstruction is performed by post-mortem EBSD of the Cu specimen.

## Introduction

Functional smart materials alongside structural materials are the corner stone of modern technology and industry. The behaviour of such materials is governed by their structural properties spanning the micro-, meso-, and macro-scale. In the case of polycrystalline materials, knowledge of their specific grain size, orientation distribution and morphology during and after manufacturing and processing (e.g. heat treatment or mechanical loading) allows to understand their resulting general thermo-mechanical behaviour^1^. In turn, these grain structural parameters are largely a consequence of the thermal treatments and mechanical processes that a polycrystalline sample has undergone in the past^2^. Thus, the overall grain structure and morphology of a sample is a signature of its thermo-mechanical history, as well as an indicator for its expected behaviour under the thermo-mechanical service conditions. Knowledge and understanding of both is hence crucial for its design and production process tailored to the prospective use case and applications.

The assessment of the grain structure of polycrystalline samples can be performed using a combination of diffraction and imaging approaches with electrons^3,4^, X-rays^5–8^ and neutrons^9–11^. Neutrons in particular, thanks to their overall superior penetration ability, are key for the non-destructive evaluation of bulk metallurgic samples that are common in engineering applications but are rather opaque for other radiation.

Here, we introduce an approach for probing and reconstructing the multi-grain structure of bulk polycrystalline materials. Based on our recently developed multi-grain indexing from Laue neutron diffraction data^12^ we present a solution, which returns the grain orientations and morphology of the individual grains in the context of the specimen which they constitute. The experimental method, which we refer to as Laue Three-Dimensional Neutron Diffraction Tomography (Laue 3DNDT), efficiently collects the diffraction signal of the sample, i.e. of all involved crystal grains, utilizing the full thermal neutron spectrum available from a specific neutron source. Due to the fact that correspondingly the wavelength involved with a particular diffraction signal is not known initially, the indexing method, that complements the measurement, is based on a forward modelling approach. Laue patterns are calculated for large sets of orientations and localizations of crystallites in the bulk sample and the data is searched for coinciding patterns. The 3D grain morphology reconstruction tackles the geometrical challenge of a non-conventional tomographic reconstruction, under the simplification that the shape of the peaks identified to belong to an individual grain is dependent only on the grain geometry. It is shown, that thus, Laue 3DNDT is able through relatively short exposures and intense computing to obtain the crystal grain positions, orientations and morphology of coarse-grained materials.

## Reconstruction

The grain indexing procedure identifies, based on forward modelling^12^, the diffraction peaks respective to a specific grain. These peaks represent projections of the individual grain with respect to the corresponding diffraction angle. In conventional tomography the projections of an object are taken in a transmission geometry and the reconstruction can be reduced to a problem in a plane geometry. The mathematical foundation to correspondingly reconstruct a 2D cross sectional slice from the 2D angular dispersive set of projections of the slice, referred to as sinogram, was provided by Radon already in 1917^13^. Accordingly, standard reconstruction programs utilize a filtered back projection algorithm based on the inverse Radon transformation to reconstruct 3D volumes as a stack of 2D cross sectional slices. However, in the case of tomographic reconstruction from diffraction data, the main challenge lies in the fact that the problem is not confined in a plane, but projections are found in 4*π*. In contrast to the continuum of projections present in conventional attenuation tomography, the diffraction spot projections are discrete and dictated by crystallography and also depend on the sample rotation in a significantly more sophisticated manner than in transmission. Thus, the reconstruction of a 3D volume from 2D diffraction projections requires an advanced approach.

In addition, the intensity of a Laue peak projection does not comply to a description as simple as the Beer Lambert law, but depends, among others, on the deflected wavelength in an undefined spectrum, the volume and shape of the grain, the structure factor of the reflecting (*hkl*) plane, the temperature of the sample, the mosaicity, the attenuation of the beam and the local efficiency of the detection system (which in a transmission experiment can be accounted for much easier). Nevertheless, the intensities of the diffraction peaks relative to individual grains enable an estimate of the grain sizes, in particular relative to each other. For the presented work, however, the latter details have been largely neglected for the 3D reconstruction, because the focus was on reconstructing the mere shape of each individual grain.

For the Laue 3DNDT a 3D parallel beam geometry is assumed. The projection directions are defined by 3D vectors along which the beam is assumed parallel, which is justified within the resolution limit of the set-up. Reconstruction is performed for each grain identified in the initial Laue 3DNDT data analysis individually. For each individual grain the corresponding peaks, their shapes and intensity distributions and the diffraction geometry, i.e. orientation of the grain and related projection angle in 3D, are well known.

**Figure 1:**
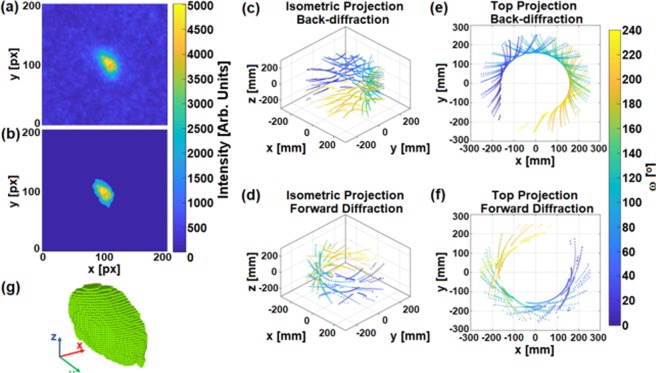
(**a**) Diffraction peak area cropped from the Laue data. (**b**) The same peak adjusted to be used as projection for tomography reconstruction. The size of the frame in which the peak is centred is decided based on the widest peak of the dataset, and the threshold value to bring the background to zero is calculated using the Otsu method^14^. (**c**–**f**) Position of the diffraction peaks from a single crystal grain in the sample reference system (SRS); (**c**,**d**) Isometric view of the diffraction peaks detected on the back-diffraction and forward diffraction detector, respectively, rotated into the SRS; (**e**,**f**) Top view of the diffraction peaks detected on the back-diffraction and forward diffraction detector rotated into the SRS. (**g**) A full grain volume (grain 1 from the Fe sample) as reconstructed with the SIRT-3D algorithm. The diffraction peak of (**a**,**b**) corresponds to this grain.

Initially, the respective 2D grain projections, have to be extracted from the measured diffraction patterns through a threshold procedure. Figure 1a,b gives an example of a peak as measured and the same peak segmented and prepared for reconstruction processing. Each individual peak area is first binarized using the Otsu method^14^. Subsequently the original peak is cropped according to its segmented shape and is centrally mounted on an empty, i.e. zero background, frame of standardized size. The standardized size has been defined by considering the largest diffraction peak of the full data set.

After this process is applied to all the segmented peaks of the Laue dataset relative to one grain the reconstruction of the individual grain morphology is performed. Figure 1c,d show the positions of the peaks in 3D for all rotations of a single grain in the sample reference system. Although the angular range of the acquisition during the experiment was 241°, a full angular coverage in the direction of rotation is possible thanks to the overlap of the forward and backward diffraction data as is illustrated in the projections to a plane of Fig. 1e,f. The reconstruction algorithm requires in addition the reconstructed volume to be centred at zero, and hence all projections have to be redefined in such reference coordinate system. Subsequently, a Simultaneous Iterative Reconstruction Technique (SIRT)^15^ algorithm is applied. A 3D version of SIRT enables the reconstruction of the 3D volumes of the grains from the 3D vector-defined projection datasets. The algorithm works by first reconstructing a volume by back projection, then forward projecting the volume in the same directions as the back projection, and iteratively minimizing the least square difference between the original dataset and the forward-projection. The ASTRA^16,17^ toolbox for tomographic reconstruction provides high performance tools for Central Processing Unit (CPU) and Graphics Processing Unit (GPU)^18^ for the latter task.

## Results

**Figure 2:**
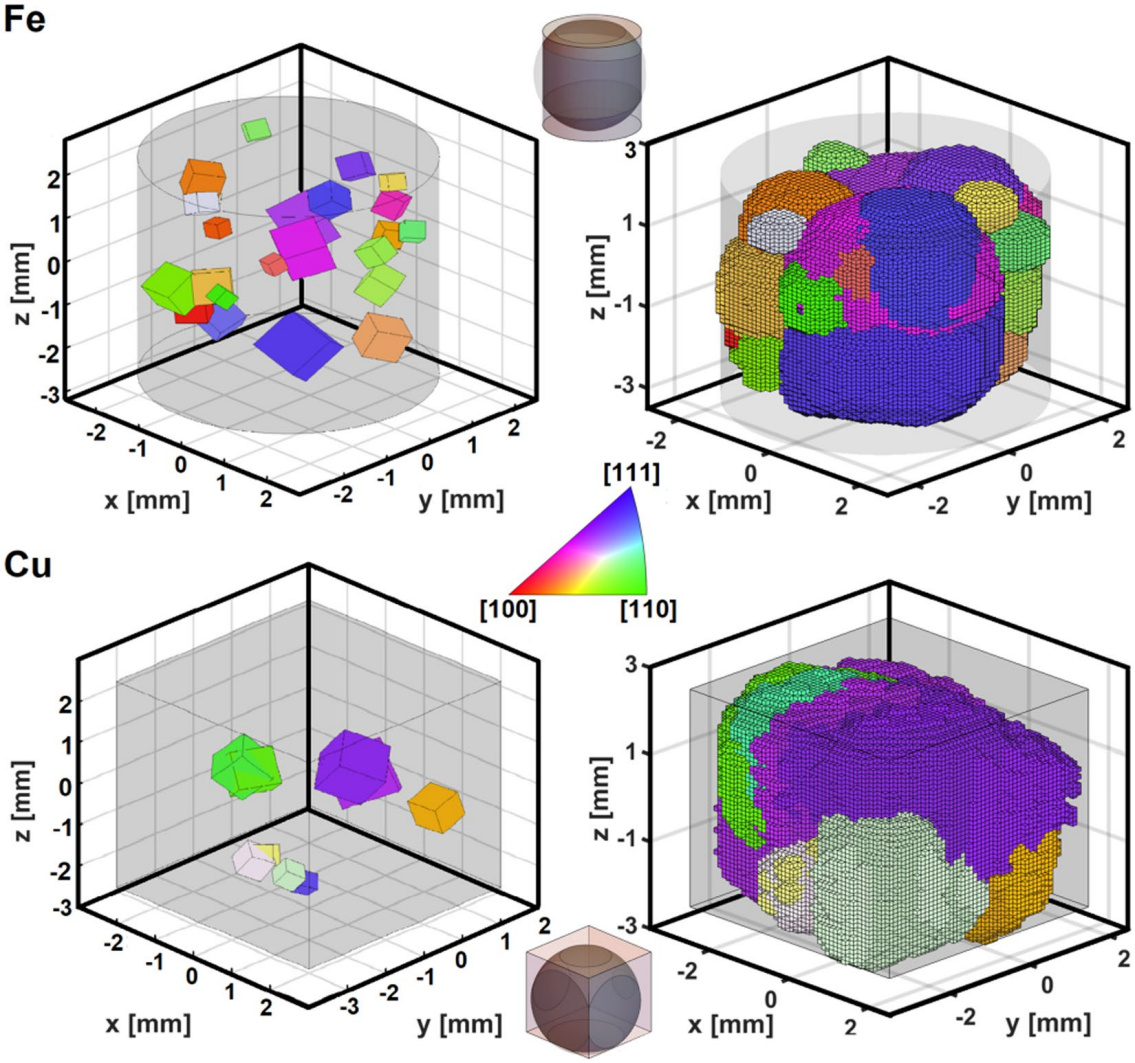
Three-dimensional orientation (top and bottom left) and morphology (top and bottom right) maps of the crystal grains of the Fe and Cu samples, respectively. The grains are colour-coded in accordance with their orientation based on the inverse pole figure colour map for a top view (positive *z*-direction) on the specimen. The shaded areas around the grains represent the shape of each sample. The small schematics given at the top and bottom of the figure show the volume area of each sample that was effectively illuminated by the neutron beam.

Iterative forward modelling^12^ returned a total number of 23 grains which were identified in the Fe sample. In the Cu sample 9 grains could be found and indexed. The reconstructed volumes of both samples, consisting of the reconstructed grains, are represented in Fig. 2 (right hand side) alongside with a schematic representation of the grains, depicted as cubes around their found centre-of-mass positions (cf. Fig. 2 left hand side), for comparison. The relative volume of the cubes in the schematic depictions was calculated, as an estimate of the respective size of the grains, using the integrated intensities of the indexed peaks. It may be seen that this relative volume estimation is in good agreement with the results obtained by the morphological reconstruction, which takes into account only the shape of the indexed reflections. This may serve as an ad-hoc validation of the 3D reconstruction of the respective grain morphologies. The grain morphology is fully reconstructed within the sample volume fully illuminated by the neutron beam. The respective probed volumes are depicted by insets in Fig. 2 through schematic illustrations of the respective gauge and sample volumes.

**Figure 3:**
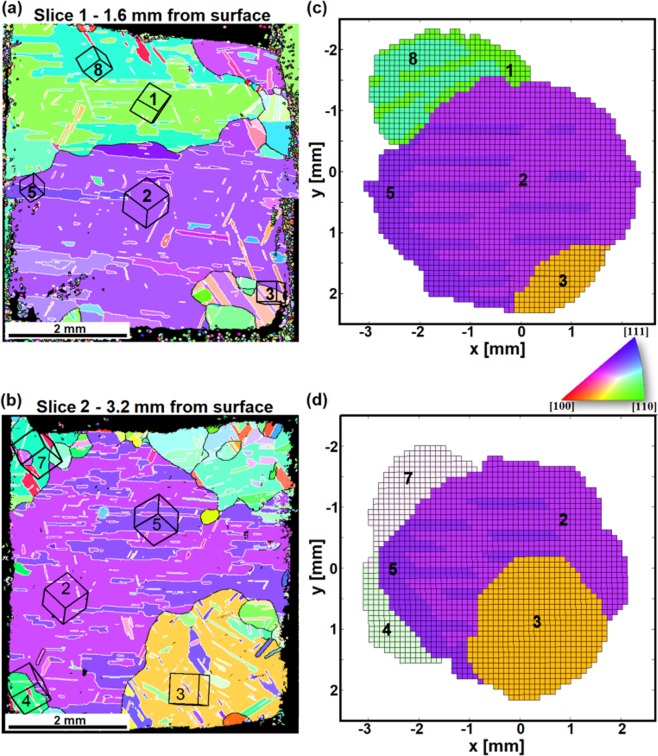
Validation of the Laue 3DNDT grain morphology reconstruction. (**a**,**b**) EBSD maps of the Cu sample taken at 1.6 mm and 3.2 mm from the surface of the sample, respectively. (**c**,**d**) Corresponding slices from the Laue 3DNDT morphology reconstruction. The grains for both the EBSD and Laue 3DNDT are numbered according to the Laue indexing order and are colour-coded in accordance with their orientation based on the inverse pole figure colour map for a top view (positive *z*-direction) on the specimen. In (**a**,**b**) the grain orientation is also schematically depicted as cubes. The black lines surrounding the grains indicate grain boundaries, while white and yellow lines show twin and double twin boundaries, respectively. Colour variation within one image (for the same grain) is attributed to the shift of the electron beam due to the large area scanned. Small colour variations for the same grains from different EBSD scans originate from slight misalignment of the sample after removing and reinserting it into the SEM sample chamber.

For actual validation of the approach, the Laue 3DNDT morphological reconstruction of the Cu sample was related to results obtained by post-mortem EBSD. Using the evaluated grain orientations and position from Laue 3DNDT, apart from the known sample geometry, it is possible to find a unique transformation that aligns the Laue 3DNDT reference system with that of the EBSD in order to identify the EBSD plane in the reconstructed sample volume. Two EBSD maps along with the identified corresponding slices from the Laue 3DNDT grain map are shown in Fig. 3. The grains reconstructed and identified in the two slices in Fig. 3 can clearly be associated and matched in shape, size and orientation, with such found in EBSD. An interesting aspect is the presence of twins indicated e.g. as grains 1 and 8 as well as 2 and 5. The EBSD map shows e.g. the existence of a big grain (grain 2 in light purple) in the central part that contains a number of twins, one of which (grain 5 in dark purple) is most pronounced. This parent-twin couple is identifiable as grains 2 and 5 in the Laue 3DNDT reconstruction, but the exact fine-structured interplay as in the EBSD cannot be resembled, due to resolution limits. However, from the neutron reconstruction it is found that the centres-of-mass of both of these two grains are in the centre of the sample having the highest calculated volume, which is well in agreement with the EBSD results. These grains, hence, simply overlap throughout almost all their reconstructed volume, which is indicated in the figure by the striped pattern. Similarly, significant grains are the orange grain (grain 3) at the right bottom corner and the green grain (grain 8) at the top along with the twin grain 1 (also in green). Overall, the reconstructed grains and their orientations could all be matched well with the grains and twining found in the EBSD (Fig. 3) and only the fine structure and grains beyond the current resolution limit of the order of 500 *μ*m were missing in our first 3D morphological Laue 3DNDT reconstruction.

## Discussion and Conclusions

The results proof the ability of Laue 3D Neutron Diffraction Tomography to provide 3D reconstructions of the grain network constituting a polycrystalline sample. The results are comparable to earlier reported low-resolution monochromatic^9^ and time-of-flight (ToF)^10^ grain mapping techniques with neutrons. The number of grains reconstructed *per volume* in the iron sample matches the one reported for the ToF method, and both exceed the one reported for neutron diffraction tomography. However, the presented white beam Laue 3DNDT technique offers significantly reduced exposure times, more than an order of magnitude as compared to the reported ToF technique. Hence, Laue 3DNDT provides superior efficiency which allows data to be taken within minutes at a medium flux source. Thus, the method offers most promising conditions for future in-situ studies exploiting the non-destructive nature of this method probing the bulk of significant sample volumes. While the effort of this technique is shifted from the exposure to the analysis, which will profit from ever increasing computing power and speed, the main challenge lies, as for comparable neutron techniques, in the remit of the spatial resolution, where improvements are desired in order to resolve the rich fine structure and full grain network as it is becoming visible in individual slices in the comparative EBSD maps. However, the relatively high flux available to this white beam technique is in favour of supporting improved spatial resolution which requires improved collimation on the one hand to improve the imaging quality of the projected grains in the diffraction peaks and improved signal-to-noise, on the other hand, enabling an improved segmentation of the grain projections from the background. Also, the continuous beam character of the measurement technique enables the utilization of state-of-the-art high-resolution imaging detectors in a regime not amenable to ToF techniques. A signature of smaller grains, which are currently not displayed in the reconstructions, are in principle found already in the current data, but they are lacking significance to allow reliable grain indexing. However, also the indexing and reconstruction software and algorithms are still subject to progress, and while our first successful attempt uses non-conventional 3D projection geometries to adapt the particularities of Laue datasets to SIRT algorithms, one could also think of using a predictive approach based on a forward model, similar or even simultaneously to the indexing procedure, to obtain each grain geometry by minimization of the difference between experiment and prediction. It is also important to point out that grain morphology reconstruction is only one of many analysis tools which are enabled once grain indexing has been performed. Thus, Laue 3DNDT complements to a high degree its X-Rays counterparts^5–8^, in particular the somewhat equivalent High Energy Diffraction Microscopy (HEDM) technique^19,20^, in extending both the grain and overall sample sizes that can be assessed.

In summary, the method of Laue three-dimensional grain morphology reconstruction was presented. Feasibility has been proven successfully through the reconstruction of 3D grain maps of a millimetre sized Fe and Cu samples returning 23 and 9 grains, respectively. The presented GPU based reconstruction tool is a powerful complement to our 3D Laue multi grain indexing algorithm^12^ enabling efficiency gains in terms of data acquisition times at comparable resolution of at least an order of magnitude compared to other reported neutron-based grain mapping methods. Validation of the presented results was conducted by means of EBSD destructive indexing analysis. The EBSD mapping was performed on the Cu sample and was found to be in good agreement with the corresponding Laue 3DNDT results. The grains matched in orientation and centre-of-mass position and in spite of the shape of the grains not being precisely reconstructed, due to current spatial resolution limitations, the sizes of the grains between Laue 3DNDT and EBSD were also in good agreement. The method thus provides excellent potential for application driven development of non-destructive 3D in-depth neutron grain mapping. The method therefore might also be considered a first step towards a new perception of Laue diffraction in general and neutron Laue diffraction in particular. The possibility to obtain macrostructural properties of oligrocrystalline samples using polychromatic Laue diffraction brings new characterisation capabilities and scientific cases for this technique in crystallography and metallurgy.

## Methods

### Samples

An ultrapure (99.98% purity) Fe cylinder, 5 mm long and 5 mm in diameter and a Cu cube with dimensions 5  ×  5  ×  5 mm^3^ were measured by means of Laue neutron diffraction. The Fe sample was obtained from a rod purchased from Goodfellow Cambridge Ltd. (Huntingdon, England), cold worked, cut, vacuum-sealed in quartz-glass capsules and heat treated at 900 °C for 20 days to grow grains with size in the hundred of microns to millimetre range. The Cu cube was cut from a Cu ingot prepared at the Paul Scherrer Institut and then heat treated at 1050 °C for 5 hours for grain growth.

### Laue 3DNDT Measurements

The data were collected at the E11 beam-port of the BER II reactor of the Helmholtz-Zentrum Berlin, Germany, using the Fast Acquisition Laue Camera for Neutrons (FALCON)^21^. FALCON consists of a forward and a back-diffraction detector. Each detector is composed of a ^6^LiF-ZnS scintillator plate with a total surface area of 400  ×  400 mm^2^ and a thickness of 250 *μ*m coupled with four iCCD cameras of 4000  ×  4000 total pixel area (100 *μ*m pixel size).

The sample-to-detector distances were 160(2) mm for both detectors with a field-of-view of 400 × 400 mm^2^ each. Hence, the angular coverage of the diffracted neutrons using both detectors is above 78% of 4*π*. The spatial resolution of the detectors of 250 *μ*m is equivalent to about 0.07° angular resolution in diffraction angles. For the measurements a continuous thermal neutron beam is used, utilizing the full available wavelength range of about 0.9  < *λ* <  3.2 Å. In both measurements the samples were fully illuminated within the effective beam size vertical dependence (≥5 mm beam diameter - cf. Fig. 2), with approximately 0.3° divergence, allowing for collection of diffraction data from all grains constituting a sample simultaneously. Diffraction data was acquired for multiple orientations of the sample with respect to rotation around a vertical axis, i.e. for a tomographic angle scan. The Fe sample was measured within a rotation range of 241° with an angular step of 1°. The Cu sample was measured within a rotation range of 241° with an angular step of 8°. The total exposure time for 241 projections of the Fe sample was 40 min and for the Cu sample for 31 projections was 7.75 min.

### Conventional EBSD

After being studied using Laue 3DNDT, the Cu sample was sliced and its microstructure was investigated by EBSD. A total number of two scan images were taken at different depths in the sample. For each scan the sample was ground with 4000 grit SiC paper and subsequently electro-polished for 10 s with a 2:1:1 (by volume) water/ethanol/phosphoric acid solution. The EBSD measurements were performed at the Paul Scherrer Institut, Switzerland, using a Zeiss ULTRA 55 field emission gun scanning electron microscope (FEG SEM), equipped with EDAX Hikari Camera operated at 20 kV in high current mode with the 120 *μ*m aperture and a step size of 10 *μ*m. The post processing of the EBSD data was done with the EDAX OIM Analysis 7.3 software.

## Data Availability

The datasets generated during and/or analyzed during the current study are available from the corresponding author on reasonable request. The Laue 3DNDT code was developed by Marc Raventós, Søren Schmidt, Stavros Samothrakitis and Camilla Buhl Larsen for MATLAB, and can be found in the GitHub repository Laue3DND,10.5281/zenodo.1553164.
